# Supplementary material for: MEG abnormalities and mechanisms of surgical failure in neocortical epilepsy
Source: Epilepsia. 2023 Jan 24;64(3):692–704. doi: 10.1111/epi.17503 (PMC10952279; doi:10.1111/epi.17503)
Supplement: Supplementary file 1 — Appendix S1 [file EPI-64-692-s001.docx]

# Supplementary

# **S1 Supplementary methods**

**S1.1 MRI acquisition parameters**

Patient MRI scans (UCLH) were acquired using standard imaging gradients with a maximum strength of 40mT m−1 and slew rate 150 T m−1 s−1. All data were acquired using a body coil for transmission, and 8 channel phased array coil for reception. Standard clinical sequences were performed including a coronal T1-weighted volumetric acquisition with 170 contiguous 1.1 mm-thick slices (matrix, 256 × 256; in-plane resolution, 0.9375 × 0.9375 mm). For healthy controls (Cardiff), T1-weighted structural data were acquired using an axial three-dimensional fast spoiled gradient recalled sequence with the following parameters: TR = 8 ms, TE = 3 ms, TI = 450 ms; flip angle = 20°; voxel size = 1 mm; field of view (FOV) ranging from 256 × 192 × 160 mm3 to 256 × 256 × 256 mm3 (anterior-posterior/left-right/superior-inferior). The T1 images were downsampled to 1.5-mm isotropic resolution.

# S2 Supplementary analysis

## S2.1 Comparison of normative maps across modalities

Normative maps of band power in healthy controls have been previously reported. In this section we compare our normative MEG maps to those derived using normative intracranial EEG data from an independent cohort of subjects. Intracranial EEG normative maps were created using intericatal data from 234 subjects. Full details of the data and generation of normative maps are provided by^20^. Although differences exist in the sample size (MEG=70, iEEG=234) across modalities, MEG typically has better spatial coverage than iEEG. For all MEG regions we have recordings for all 70 healthy controls. However, for iEEG, coverage within each region is determined by the subject electrode placements. That is, cortical temporal regions may have better coverage than other cortical regions as temporal lobe epilepsy is one of the most common types of focal epilepsy. To quantify the similarities across modalities we used Pearson correlations. Figure S1 illustrates the relationship between the normative maps across modalities. We see strong associations between MEG and iEEG normative maps in the delta (Pearson R=0.61), alpha (Pearson R=0.78), and beta (Pearson R=0.67) frequency bands. Weaker correlations are reported for theta (Pearson R=0.36), and gamma (Pearson R=-0.2) frequency bands. MEG recordings were collected with controls at rest with eyes closed. Conversely, iEEG recordings were collected at rest with eyes open. Our differences found in theta may be attributed to alpha de-synchronisation, a phenomenon commonly observed between eyes-open and closed recordings. We postulate that differences between modalities gamma may be due to the low signal to noise ratio commonly attributed to higher frequencies.


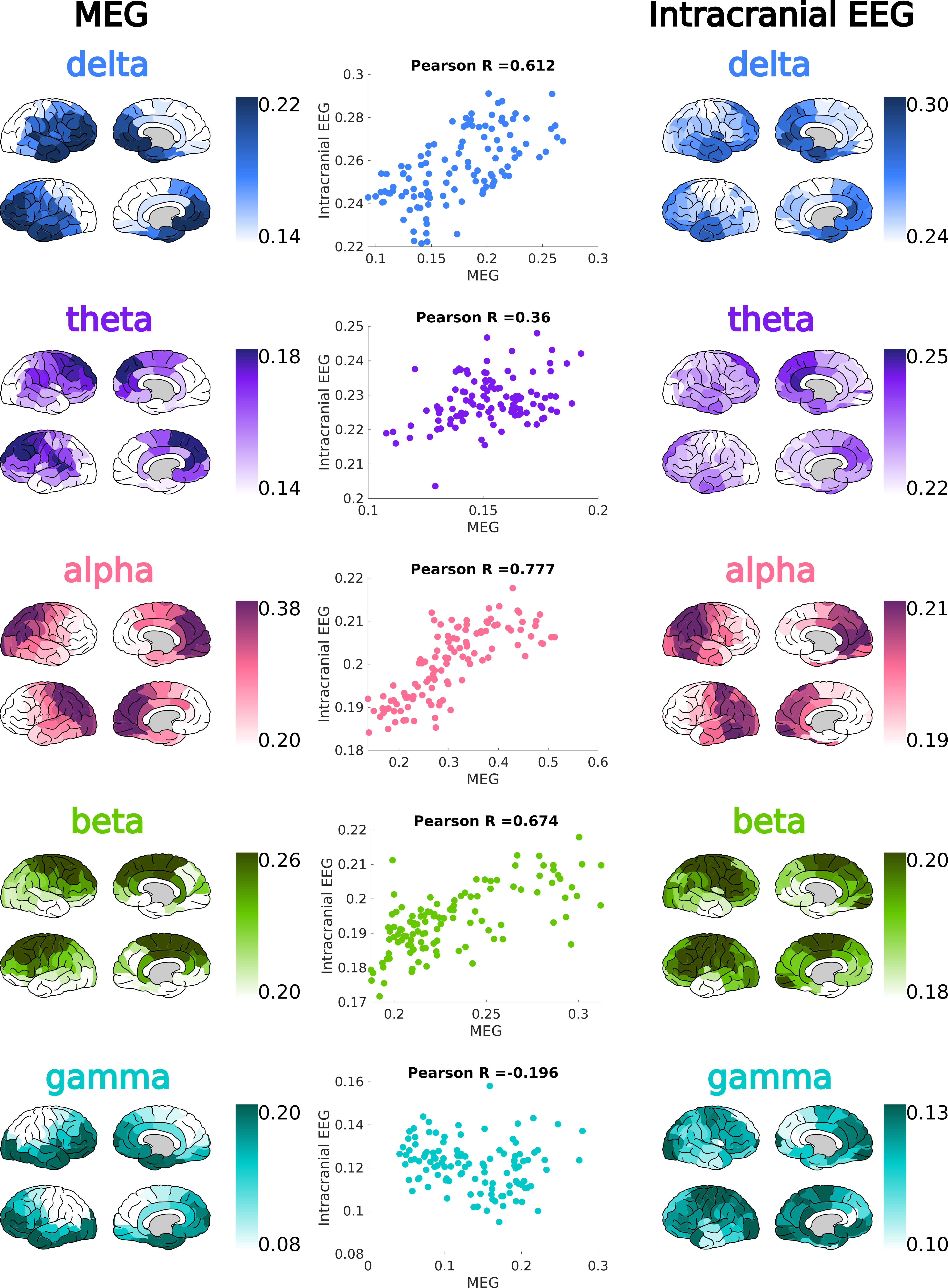


**Figure S1: Comparisons of normative maps derived using MEG and intracranial EEG data.** Normative maps for MEG **(left column)** and iEEG **(right column)** are provided for all five frequency bands. Scatterplots **(centre column)** depict the associations between the two modalities. Pearson correlations quantify the association between the two modalities. Note the difference in scale between the MEG and iEEG relative band power contributions.

## S2.2 Comparison of mechanisms

We proposed three mechanisms hypothesised the relate to surgical outcome. Here we used Pearson correlation to assess and quantify the similarity of these mechanisms. The results of our comparison are illustrated in figure S2. Weak associations exist between mechanism 1 and 3 and mechanisms 2 and 3 with Pearson correlations of R=0.388, and R=-0.373 respectively. A stronger association exists between mechanisms 1 and 2, R=-0.638, yet variability still exists, suggesting complementary information.


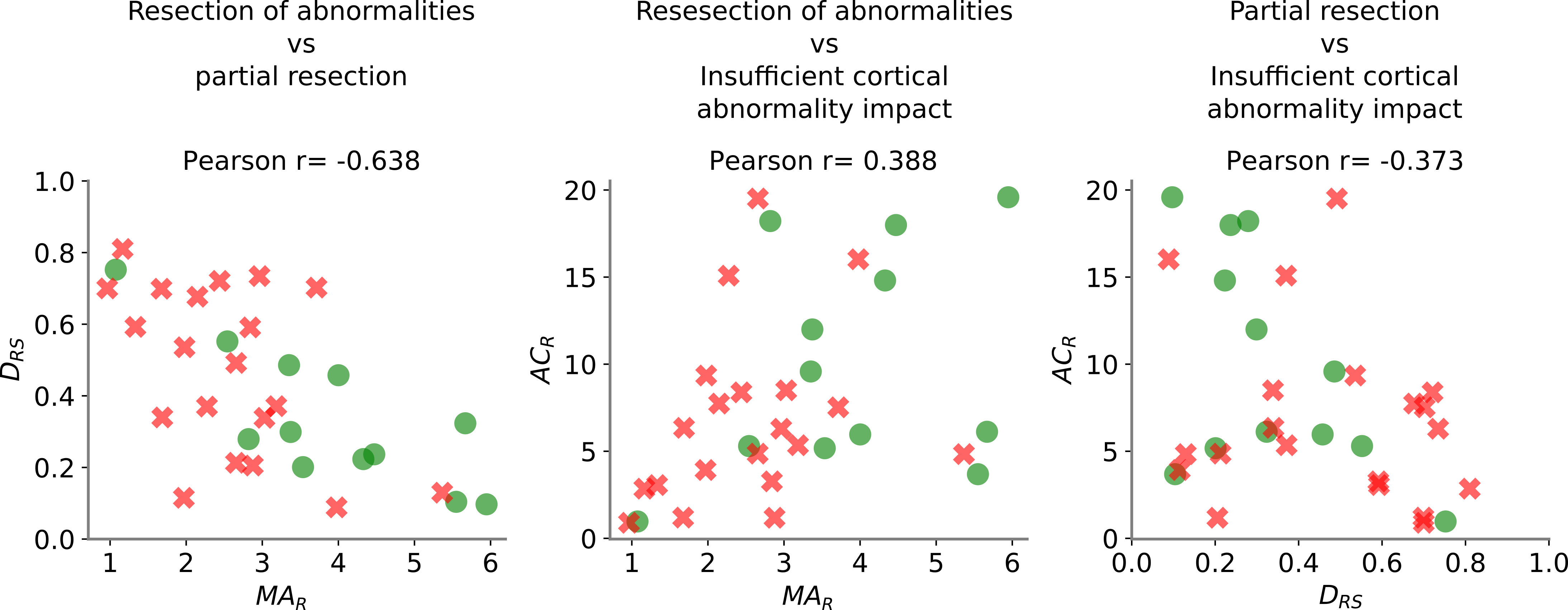


**Figure S2: Similarity of mechanisms.** Scatterplots illustrate the relationship between each pair of mechanisms. Pearson correlations quantify the similarity between each pair of mechanisms. Each datapoint is an individual patient. Good outcome patients (ILAE 1) are depicted with green circles, whereas bad outcome patients (ILAE 2+) are depicted with red crosses.

## S2.3 Consistency of results to variations in parcellation scheme and epoch

In the main text we investigated mechanisms of surgical failure using only one parcellation with 114 cortical regions, and one 70-second segment (epoch). In this section we assess the robustness of our mechanisms to changes in parcellation scheme and epoch. To ensure our findings are robust we repeated the analysis, first for different resolution parcellation schemes, and second for an independent epoch of data. Figure S3 illustrates the AUC for each mechanism when the resolution of the parcellation scheme changes (i.e. replication of the AUC in figure 5). The Lausanne parcellation scheme at four different resolutions (68, 114, 219 and 448 neocortical regions of interest) was compared. For all three mechanisms, only negligible differences exist between the parcellation schemes, suggesting that the mechanisms are invariant to changes region size. Time varying fluctuations in mechanism measures were quantified using Pearson correlations. The consistency of mechanism measures for all patients are illustrated in figure S4. All three mechanisms demonstrate strong consistency across epochs with correlation coefficients of R=0.93, R=0.72, and R=0.97 for mechanisms 1,2, and 3 respectively.


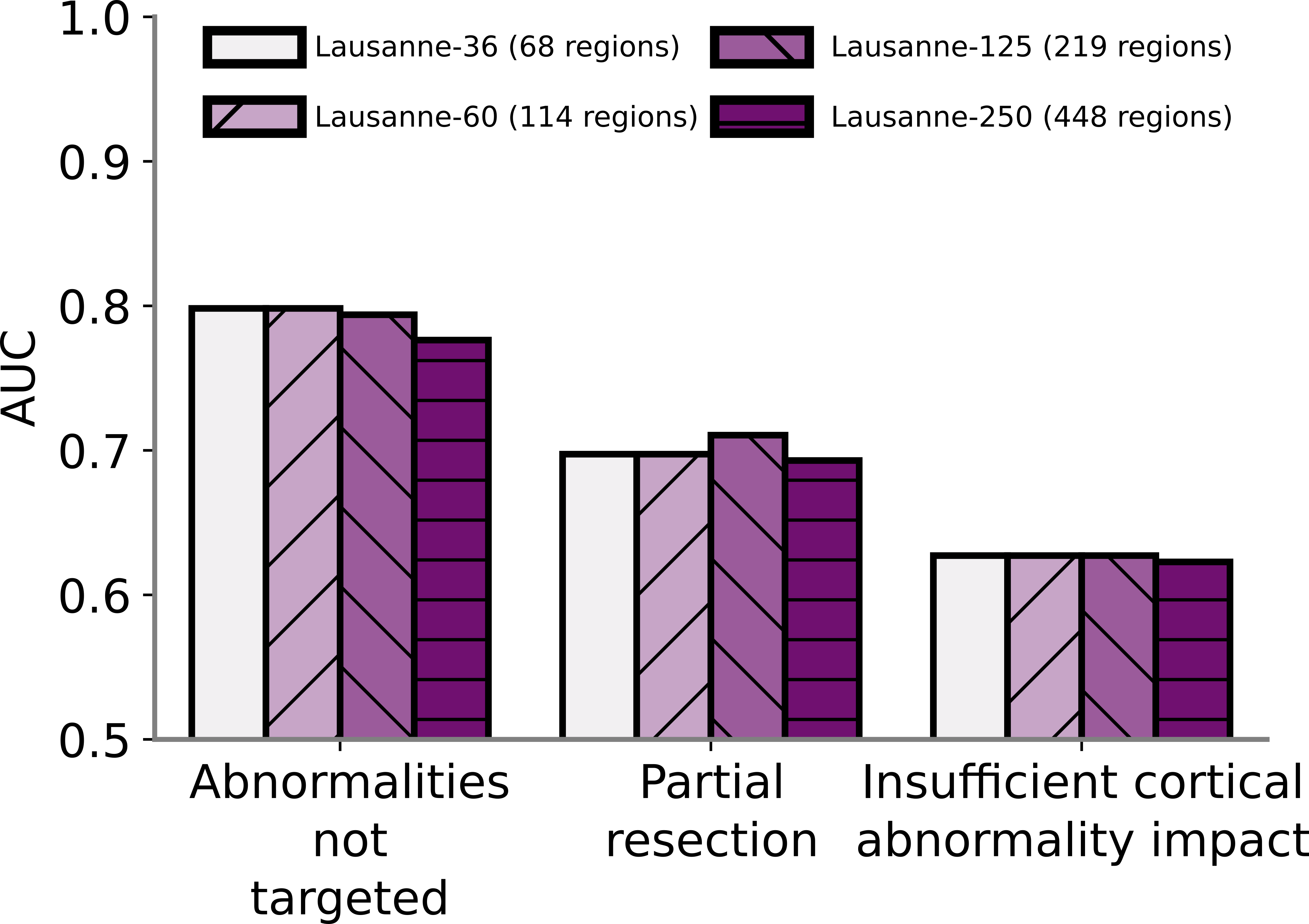


**Figure S3: Robustness to changes in parcellation scheme.** Each bar corresponds to the AUC obtained when comparing surgical outcome groups for each mechanism. Four different sub-parcellations of the Lausanne atlas are compared; Lausanne-36 (68 ROIs), Lausanne-60 (114 ROIs), Lausanne-125 (219 ROIs), Lausanne-250 (448 ROIs). Each parcellation is illustrated with a different color and texture. Note 31 out of 32 patients were compared as the resection cavity of one patient was too small to identify using the most coarse parcellation.


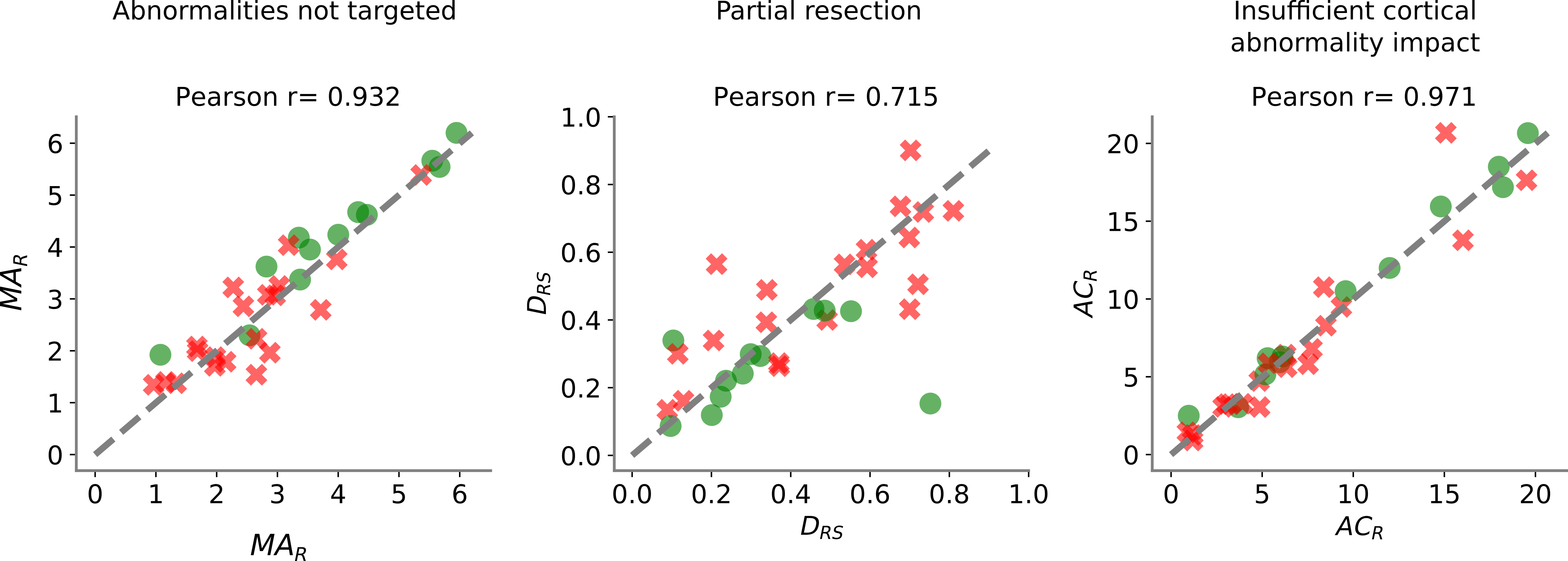


**Figure S4: Robustness to changes in epoch.** Scatterplots illustrate the consistency of mechanism measures across two epochs of data. Two distinct seventy second windows were selected for each patient and compared. Pearson correlations quantify the robustness of mechanisms to changes in epoch. Each datapoint is an individual patient. Good outcome patients (ILAE 1) are depicted with green circles, whereas bad outcome patients (ILAE 2+) are depicted with red crosses. Dashed line indicates the identity line.

## S2.4 Analysis of patients with subcortical resections

Temporal lobe epilepsy is the most common type of focal epilepsy, yet patients with resections containing subcortical structures such as the hippocampus and amygdala were omitted from the analysis in the main text. Our mechanisms of surgical failure are quantified using interictal MEG recordings. Although MEG has good spatial coverage of neocortical regions, resting-state recordings of deep brain allocortical structures such as the hippocampus may be unreliable. As it has been extensively shown in the literature, allocortical structures play a cruical role in temporal lobe epilepsy. To that end, we hypothesised that our mechanisms of surgical failure would not perform well for TLE patients with subsequent resection to allocortex as subcortical coverage is missing. We analysed a cohort of 19 TLE patients (10 ILAE 1 and 9 ILAE 2+), assessing whether any of the proposed mechanisms discriminate surgical outcome groups. Results of this analysis are illustrated in figure S5. We show that as hypothesised, no single mechanism discriminates surgical outcome groups beyond chance.

In figure S5 (left panel) we see that even in ILAE 1 patients the average resection abnormality is low ($<3$ for most ILAE 1 patients, c.f. figure 5 in main text; $>4$ for most ILAE 1 patients). We postulate this is because the resected regions in these patients are resected only to gain access to deeper epileptogenic tissues such as the hippocampus, rather than being epileptogenic and abnormal themselves. Without any hippocampal abnormalities present in our analysis it is therefore unsurprising that our measure for mechanism 1 does not discriminate outcome groups.


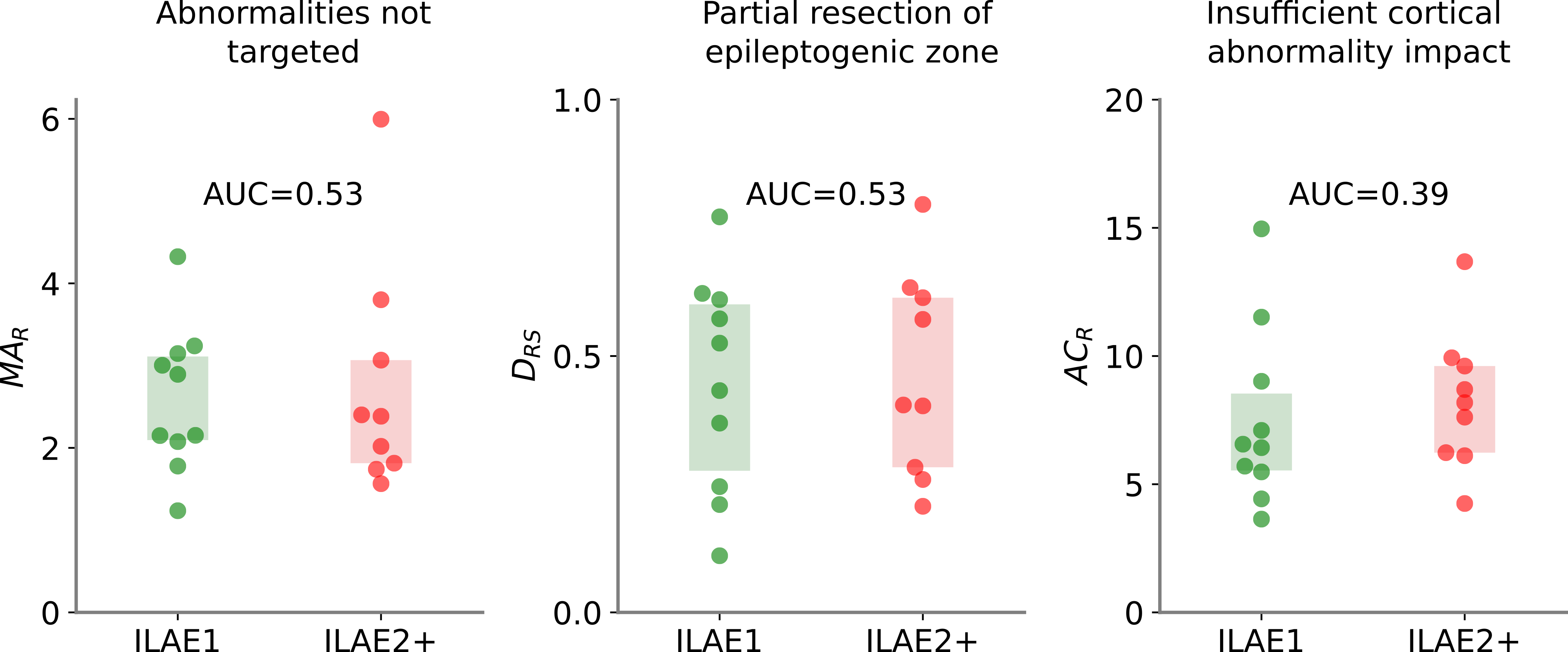


**Figure S5: Surgical outcome separability of each mechanism at the group level for a cohort of temporal lobe epilepsy patients** Boxplots illustrate how well each mechanism discriminates surgical outcome groups. For each mechanism, the AUC is calculated along with corresponding p-value using a one-tailed Mann-Whitney U test. Good outcome patients (ILAE 1) are depicted in green, and bad outcome (ILAE 2+) in red. **(**)** corresponds to statistical significance at the 1% level.

## S2.5 Table of patient data

|  | **Controls**  **(1)** | **ILAE 1**  **(2)** | **ILAE 2+**  **(3)** | **Test statistic** |
| --- | --- | --- | --- | --- |
| N | 70 | 12 | 20 |  |
| Age  (mean, SD)  [Min, Max] | 26.9 (6.9)  [19, 55] | 32.3 (10.7)  [22,54] | 32.3 (11.3)  [18,60] | $P_{1,2}=0.058, t=2.085$  $P_{1,3}=0.057, t=2.003$  $P_{2,3}=0.807, t=-0.247$ |
| Sex  (Male, Female) | 27, 43 | 7, 5 | 10, 10 | $P_{1,2}=0.334, \chi^{2}=0.935$  $P_{1,3}=0.510, \chi^{2}=0.434$  $P_{2,3}=0.927, \chi^{2}=0.008$ |
| Age onset  (mean, SD) | N/A | 9.7 (7.6) | 12.3 (6.8) | $P_{2,3}=0.356, t=0.945$ |
| Epilepsy duration  (mean, SD) | N/A | 23.6 (10.0) | 20.0 (8.8) | $P_{2,3}=0.333, t=-0.992$ |
| Resection hemisphere  (Left, Right) | N/A | 6, 6 | 11, 9 | $P_{2,3}=0.927, \chi^{2}=0.008$ |

***Table S2.5: Summary of patient and control data demographics.*** *Statistical comparisons are made using two-sample t-tests for continuous variables and* $\chi^{2}$ *tests for categorical variables. Two-tailed statistical tests were performed as no clear hypothesis of direction was provided.*

**S2.6 Frequency band contribution in patient abnormality maps**

We compared the distribution of frequency band contribution towards maximum abnormality maps across all 32 neocortical patients. The results, figure S6, show heterogeneity across the cohort. Stacked bar plots illustrate the differences between across patients, with each bar corresponding to a patient and the proportion of each colour within the bar corresponding to the contribution of each frequency band. In some patients a single frequency band dominate the abnormality map, with other frequencies not contributing at all. Contrarily, in other patients the contribution of each frequency band is more homogenous. This variability within and between patients demonstrates that when no clear hypothesis of frequency band is provided a framework invariant to frequency may be of value.


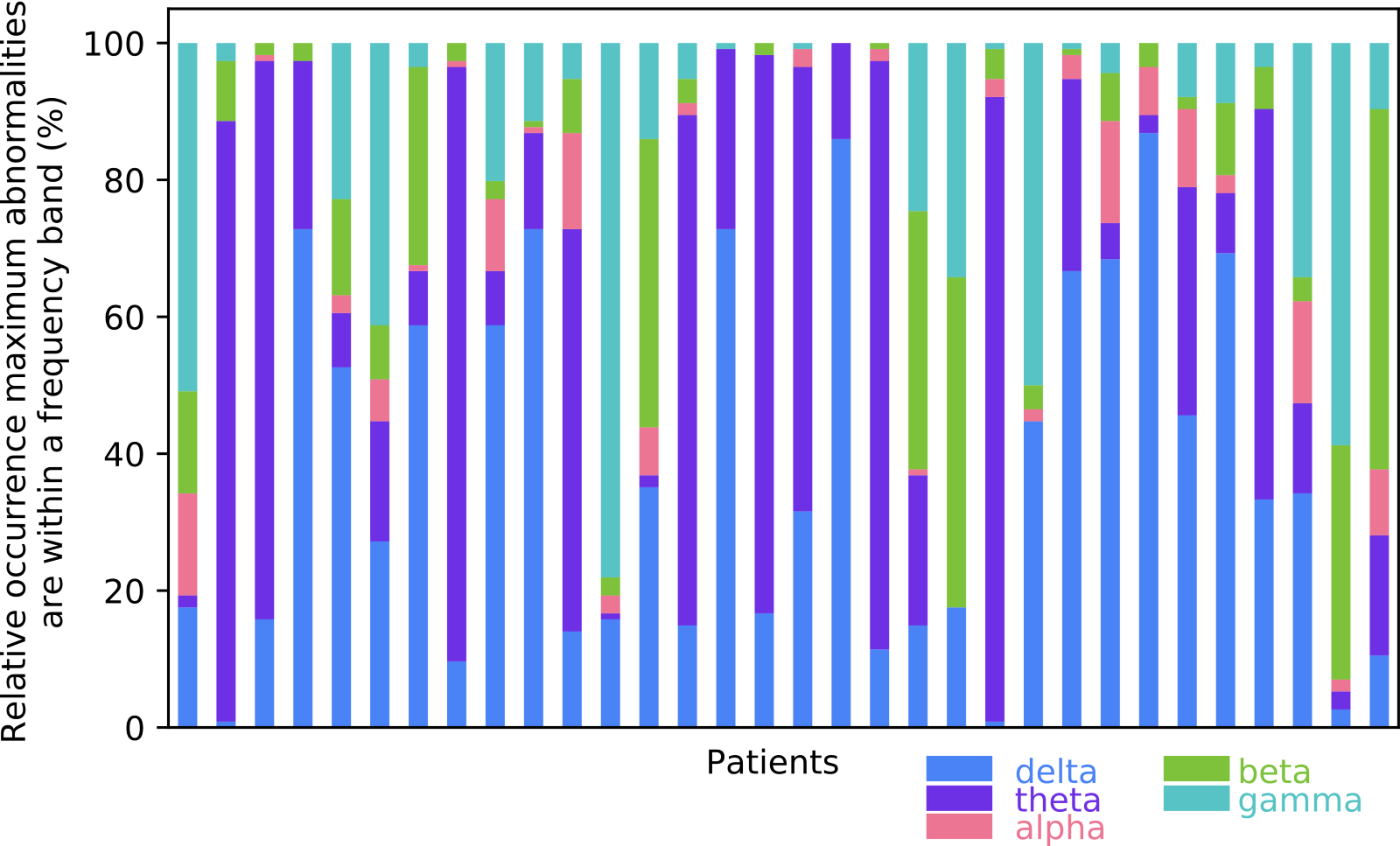


***Figure S2.6: Summary of how often maximum regional abnormalities within patients occurred within patients for each of the five frequency bands.*** *Stacked bars show the proportion of necortical regions with maximum abnormality in each frequency band. The more a frequency band contributes to the maximum abnormalities the more the corresponding colour makes up the total of the bar. Each bar corresponds to an individual patient’s abnormality frequency band profile.*

**S2.7 Patient markers of surgical failure and clinical information**

| Patient | MAR | DRS | ACR | Age | Sex | Side | Resection site | Pathology | ILAE |
| --- | --- | --- | --- | --- | --- | --- | --- | --- | --- |
| 1 | 1.165 | 0.81 | 2.853 | 32 | M | R | P | FCD II.B | 3 |
| 2 | 2.964 | 0.734 | 6.293 | 39 | M | R | F | G | 5 |
| 3 | 5.365 | 0.13 | 4.837 | 19 | F | R | P | FCD II.A | 4 |
| 4 | 5.947 | 0.097 | 19.584 | 25 | M | R | O | NA | 1 |
| 5 | 2.654 | 0.213 | 4.864 | 20 | M | L | O P | G | 4 |
| 6 | 4.001 | 0.458 | 5.966 | 29 | M | L | T | G | 1 |
| 7 | 1.969 | 0.115 | 3.923 | 32 | F | L | T | G | 4 |
| 8 | 1.686 | 0.34 | 6.344 | 24 | F | L | T | NA | 5 |
| 9 | 3.535 | 0.201 | 5.175 | 25 | F | L | F | FCD II.B | 1 |
| 10 | 2.876 | 0.205 | 1.183 | 43 | F | L | F | G | 5 |
| 11 | 3.184 | 0.371 | 5.347 | 45 | M | R | F | G | 4 |
| 12 | 0.963 | 0.7 | 0.893 | 38 | F | L | F | FCD II.A | 5 |
| 13 | 2.44 | 0.721 | 8.382 | 23 | M | L | F | FCD II.A | 4 |
| 14 | 2.82 | 0.279 | 18.216 | 29 | F | R | O P | TSL | 1 |
| 15 | 4.472 | 0.237 | 17.989 | 40 | M | R | F | FCD II.B | 1 |
| 16 | 2.147 | 0.677 | 7.737 | 31 | F | R | F | FCD II.B | 2 |
| 17 | 1.978 | 0.536 | 9.348 | 53 | F | L | F | DNT | 5 |
| 18 | 2.841 | 0.591 | 3.272 | 36 | F | L | F | NA | 5 |
| 19 | 5.548 | 0.104 | 3.684 | 28 | M | L | F | FCD II.B | 1 |
| 20 | 4.328 | 0.223 | 14.806 | 29 | M | L | F | GL | 1 |
| 21 | 1.333 | 0.593 | 3.058 | 29 | M | R | F | FCD II.A | 4 |
| 22 | 3.977 | 0.089 | 16.023 | 33 | M | R | F | CG | 3 |
| 23 | 3.029 | 0.339 | 8.5 | 18 | M | L | F | NA | 2 |
| 24 | 2.541 | 0.552 | 5.299 | 44 | F | R | P | DNT | 1 |
| 25 | 5.668 | 0.323 | 6.119 | 28 | F | L | F | FCD II.A | 1 |
| 26 | 1.675 | 0.699 | 1.191 | 60 | F | L | F | FCD II.B | 2 |
| 27 | 1.074 | 0.752 | 0.969 | 54 | F | R | T | CAV | 1 |
| 28 | 2.657 | 0.492 | 19.512 | 23 | F | L | F | G | 2 |
| 29 | 2.273 | 0.37 | 15.082 | 28 | M | R | P | FCD II.B | 3 |
| 30 | 3.352 | 0.486 | 9.58 | 22 | M | L | O | FCD II.A | 1 |
| 31 | 3.713 | 0.702 | 7.523 | 21 | M | R | F | FCD II.A | 3 |
| 32 | 3.372 | 0.299 | 11.995 | 47 | M | R | F | G | 1 |

***Table S2.7: Details of patients studied with specific markers of surgical failure and clinical data.*** *Patients 1-4 correspond to the same four patients in the main text.*
*Abbreviations:* ***MAR****: Mean abnormality of resection,* ***DRS****: Discriminability of resected and spared,* ***ACR****: Abnormality contribution of resection.* ***Resection site****; F: Frontal, T: Temporal, P: Parietal, O: Occipital.* ***Pathology****; FCD: Focal cortical dysplasia (grade), G: Gliosis, NA: Nothing abnormal detected, TSL: Tuberous Sclerosis, DNT: Dysembryoplastic neuroepithelial tumour, CG: Cortical Gliosis, GL: Glioma, CAV: Cavernoma.*

**S2.8 Table of group results using different frequency bands for abnormality mapping**

|  | Mechanism 1 | Mechanism 2 | Mechanism 3 |
| --- | --- | --- | --- |
| Delta | 0.804 (0.002) | 0.692 (0.038) | 0.679 (0.049) |
| Theta | 0.538 (0.370) | 0.363 (0.904) | 0.492 (0.539) |
| Alpha | 0.696 (0.035) | 0.621 (0.134) | 0.608 (0.160) |
| Beta | 0.633 (0.110) | 0.542 (0.356) | 0.6 (0.180) |
| Gamma | 0.404 (0.82) | 0.433 (0.740) | 0.513 (0.461) |
| Maximum | 0.796 (0.003) | 0.675 (0.053) | 0.642 (0.096) |

***Table S2.8: Summary of the discriminatory power of each marker using different frequency bands.*** *AUC scores (p-values) of the cohort wide surgical outcome discriminatory power of each marker when abnormalities are selected based on a particular frequency band or the region maximum across frequency bands.*
